# Improving Physicochemical Stability of Quercetin-Loaded Hollow Zein Particles with Chitosan/Pectin Complex Coating

**DOI:** 10.3390/antiox10091476

**Published:** 2021-09-16

**Authors:** Muhammad Aslam Khan, Chufan Zhou, Pu Zheng, Meng Zhao, Li Liang

**Affiliations:** 1 State Key Laboratory of Food Science and Technology, Jiangnan University, Wuxi 214122, China; aslamkhan255@yahoo.com (M.A.K.); chufan.zhou@mail.mcgill.ca (C.Z.); 2 School of Food Science and Technology, Jiangnan University, Wuxi 214122, China; 3 School of Biotechnology, Jiangnan University, Wuxi 214122, China; zhengpu@jiangnan.edu.cn; 4 State Key Laboratory of Biobased Material and Green Papermaking, Qilu University of Technology, Shandong Academy of Sciences, Jinan 250353, China; 2001zhaomeng@163.com

**Keywords:** hollow zein particle, chitosan, pectin, quercetin, coating

## Abstract

Hollow nanoparticles are preferred over solid ones for their high loading capabilities, sustained release and low density. Hollow zein particles are susceptible to aggregation with a slight variation in the ionic strength, pH and temperature of the medium. This study was aimed to fabricate quercetin-loaded hollow zein particles with chitosan and pectin coating to improve their physicochemical stability. Quercetin as a model flavonoid had a loading efficiency and capacity of about 86–94% and 2.22–5.89%, respectively. Infrared and X-ray diffraction investigations revealed the interaction of quercetin with zein and the change in its physical state from crystalline to amorphous upon incorporation in the composite particles. The chitosan/pectin coating improved the stability of quercetin-loaded hollow zein particles against heat treatment, sodium chloride and in a wide range of pH. The complex coating protected quercetin that was encapsulated in hollow zein particles from free radicals in the aqueous medium and enhanced its DPPH radical scavenging ability. The entrapment of quercetin in the particles improved its storage and photochemical stability. The storage stability of entrapped quercetin was enhanced both at 25 and 45 °C in hollow zein particles coated with chitosan and pectin. Therefore, composite hollow zein particles fabricated with a combination of polysaccharides can expand their role in the encapsulation, protection and delivery of bioactive components.

## 1. Introduction

Hollow zein particles have been fabricated by wrapping sodium carbonate (Na_2_CO_3_) nanoprecipitate as sacrificial templet with zein in ethanol–water binary mixture followed by antisolvent precipitation [1]. Hollow particles in the loading and controlled release of bioactive components were preferred over their solid counterpart for more surface area and low density [2], but proteinaceous nature limits their utility as an efficient delivery system due to destabilization around pI (5–6.5), presence of counterion and high temperature-induced denaturation [3,4]. Numerous strategies have been adopted to overcome instability issues of zein particles, for instance, coating with proteins [5,6], polysaccharides [7] and lipids [8]. Pectin is an anionic biodegradable polymer found in the plant cell wall and mainly made up of methyl esterified 1-4 linked α-D-galacturonic acid and 1-2-linked α-L-rhamnopyranose. Composite hollow zein particles with casein and pectin were developed by heating at 80 °C and 6.2 pH for 1 h to attain outstanding stability under simulated gastrointestinal conditions. Still, these composite particles were limited only to encapsulate and deliver heat-sensitive bioactives [9].

Chitosan-coated solid zein particles were fabricated through hydrophobic, hydrogen and van der Waals interactions at pH 4, improving the entrapment, photo/thermal protection and controlled release of bioactive components [10,11,12]. Chitosan, a N-acetyl-D-glucosamine and D-glucosamine β1-4 linked cationic polymer, is obtained by deacetylation of chitin and considerably used for the stabilization of delivery systems [13]. However, the deprotonation of the amine groups of chitosan above pk_a_ (~pH 6.5) reduces charge density, and chitosan competes with counterions with the increase in ionic strength, heading towards destabilization [12,14]. It has been reported that pectin imparted good pH and heating stability to zein core–shell nanoparticles, but the endurance for increasing ionic strength was extremely weak [15,16]. Chitosan and pectin could form polyelectrolyte complexes via electrostatic interaction [17], which was used to improve the physiochemical stability of nanoliposome [18]. Therefore, a combination of chitosan and pectin may synergistically and resourcefully bear variation in pH, temperature and counterions.

Quercetin is a flavonoid with antioxidant, anticarcinogenic, antiviral and anti-inflammatory properties. Its low solubility in water and chemical instability have been addressed through biopolymer-based nano/micro-delivery systems for the application in functional foods [19,20]. In the current work, composite hollow zein particles were fabricated with chitosan-pectin complex coating for the encapsulation and protection of quercetin. The particles were characterized for size, ζ-potential and loading efficiency of quercetin. The lyophilized samples of quercetin-loaded composite particles were analyzed with infrared and X-ray diffraction techniques. Moreover, the particle dispersions were subject to varying pH, ionic strength and temperature conditions to assess physical stability. Finally, the antioxidant activity, photochemical and storage stability of quercetin encapsulated in composite hollow zein particles were examined to judge the protective effects of the particles. This study focused on the fabrication of composite hollow zein particles with improved physical stability and better protective effect on flavonoids through tailoring a complex polysaccharide interfacial layer.

## 2. Materials and Methods

### 2.1. Materials

Zein from corn (~98%) was purchased from J&K Chemical Ltd. (Shanghai, China). Sodium carbonate (Na_2_CO_3_, ~99.8%) was purchased from Sinopharm Chemical Reagent Co., Ltd. (Shanghai, China). Pectin from citrus peel was purchased from Shanghai Sangong Bioengineering Co., Ltd. (Shanghai, China). Chitosan (low molecular weight, 50–190 KDa) and quercetin (≥95%, HPLC) were purchased from Sigma-Aldrich Co. (Shanghai, China). Ultra-pure water obtained using a Milli-Q direct water purification system equipped with Quantum TEX column (Molsheim, France) was used throughout all the experiments. 

### 2.2. Preparation of Blank and Quercetin-Loaded Hollow Zein, Hollow Zein-Chitosan, and Hollow Zein-Chitosan/Pectin Particles

Hollow zein (HZ) particles were fabricated with sodium carbonate sacrificial templet by mixing 1.75 mL of absolute ethanol and 2.5 mL of 50 mg/mL zein in 70% (*v/v*) ethanol–water binary mixture with 0.75 mL of 0.5, 1 and 2 (*w/v*%) Na_2_CO_3_ aqueous solution under magnetic stirring at 1000 rpm for 1 min followed by adding to a 20 mL ultra-pure water [1,21]. The HZ particles were mixed with 0.5, 1 and 2 mg/mL chitosan in 1% (*v/v*) acetic acid at a 1:1 volume ratio under stirring at 1000 rpm for 30 min. Ethanol in the particle dispersion was removed under vacuum with a rotary evaporator RE-52C (Shanghai Tianheng Instrument Co. Ltd., Shanghai, China) at 35 °C for 30 min. Unstable particles were separated by centrifugation the particle dispersion at 2000× *g* for 15 min; the supernatant was then centrifuged at 15,000× *g* for 30 min to remove unabsorbed chitosan, and the precipitate was redispersed in an equal volume of distilled water to obtained chitosan-coated hollow zein (HZ-chi) particles. The aqueous solutions of pectin at 0.01, 0.025, 0.05 and 0.1 mg/mL were added to the dispersion of HZ-chi particles under stirring for 30 min at pH 4–4.5 to obtain pectin- and chitosan-coated hollow zein (HZ-chi/pec) particles. The quercetin-loaded hollow particles were prepared by adding 2, 3, 4 and 5 mg of quercetin in 50 mg/mL zein stock solution in ethanol–water binary mixture corresponding to 100, 150, 200 and 250 µg/mL of quercetin in the final particle dispersion. 

### 2.3. Characterization of Particles 

#### 2.3.1. Particle Size and ζ-Potential 

Samples were diluted by 200 folds with distilled water and measured at 25 °C and analyzed on a NanoBrook Omini particle size analyzer (Brookhaven Instrument, New York, NY, USA) at a scattering angle of 90°. NNLS function was used to acquire the size distribution, while phase analysis light scattering (PALS) was employed to estimate the ζ-potential. Samples were prepared in triplicates for each measurement.

#### 2.3.2. Loading Efficiency and Loading Capacity of Quercetin

Quercetin-loaded hollow zein and hollow zein-chitosan particles were centrifuged at 2000× *g* for 15 min to remove unencapsulated quercetin, and the supernatant and samples were diluted 50-fold in ethanol for the measurement of quercetin absorption at a λ_max_ of 373 nm using a UV1800 UV-Vis spectrophotometer (Shimadzu Corporation, Tokyo, Japan) with a standard curve constructed from 1 to 20 μg/mL of quercetin dissolved in ethanol (a correlation coefficient of 0.999, Figure S4), adopting the method of Wang et al. [22]. Samples were prepared in triplicates for each measurement. Loading efficiency and capacity of quercetin in the particles were determined by the following equations.

(1) $$\mathrm{Loading}\;\mathrm{efficiency}\;(\%)=\frac{\;\mathrm{Quercetin}\;\mathrm{in}\;\mathrm{supernatant}\;}{\mathrm{Total}\;\mathrm{added}\;\mathrm{quercetin}}\;\times \;100$$

(2) $$\mathrm{Loading}\;\mathrm{capacity}\;(\%)=\frac{\;\mathrm{Quercetin}\;\mathrm{entrapped}\;\mathrm{in}\;\mathrm{particles}\;(\mu g)\;}{\mathrm{Zein}\;\mathrm{and}\;\mathrm{chitosan}\;(\mu g)}\;\times \;100$$

#### 2.3.3. Microstructural Analysis

Lyophilized particles were mounted to the surface of double-sided carbon tape and coated with a thin layer of gold. The morphology of particles was observed with a Hitachi SU8010 FE-SEM (Hitachi, Co., Tokyo, Japan) operated at an accelerating voltage of 8 kV. 

#### 2.3.4. Infrared Spectroscopy (IR)

Samples were freeze-dried and pressed into a transparent pellet with KBr, Infrared spectra of particles and raw materials were collected in the range of 400–4000 cm^−1^ on Nicolet™ iS™ 10 FT-IR Spectrometer (Thermo Fisher Scientific, Waltham, MA, USA) at a 4 cm^−1^ resolution and 16 scans. 

#### 2.3.5. X-ray Diffraction (XRD)

A Bruker D2 PHASER (Brucker, Odelzhausen, Germany) X-ray diffractometer, operated at 30 kV, 10 mA was used to obtain X-ray diffractograms of quercetin, zein, chitosan, pectin and quercetin-loaded hollow zein particles coated with chitosan and pectin. The data were collected over an angular range from 5° to 40° 2θ in continuous mode using step size and time of 0.02° and 5 s, respectively.

### 2.4. Stability Assessment of Particles under Stressed Condition

#### 2.4.1. Sodium Chloride Stability

Freshly prepared quercetin-loaded HZ, HZ-chi and HZ-chi/pec particles were exposed to 50, 100, 200, 300, 400, 500 mM sodium chloride under continuously mixing for 30 min followed by a 5 min rest [8]. Particle size and ζ-potential were measured, as mentioned above in Section 2.3.1.

#### 2.4.2. pH Stability

The pH values of quercetin-loaded HZ, HZ-chi and HZ-chi/pec particles were adjusted from 2 to 9 with 0.1 mM hydrochloric acid and sodium hydroxide and then continuously stirred for 30 min [8]. Particle size and ζ-potential were analyzed by diluting them in pH-adjusted Milli-Q water [23].

#### 2.4.3. Temperature Durability

Quercetin-loaded HZ, HZ-chi and HZ-chi/pec particles were incubated in a water bath at 30, 40, 50, 60, 70, 80 and 90 °C for 30 min and evaluated for particle size and ζ-potential [8]. 

### 2.5. Antioxidant Activity

ABTS and DPPH assays of quercetin-loaded HZ and HZ-chi/pec particles were estimated according to the method of Dong et al. and Pan et al. [24,25]. In brief, equal volumes of potassium persulfate (2.6 mM) and ABTS (7.4 mM) were mixed and allowed to react and generate ABTS^+^ for 12 h in the dark. After the diluted ABTS^+^ solution with an absorbance of 0.7 at 734 nm was mixed with samples at a volume ratio of 2:1 in the dark for 6 min, the absorbance was measured at 734 nm on Synergy H1 Microplate Reader (BioTek Instruments, Inc., Winooski, VT, USA). Likewise, after 0.1 mM DPPH in ethanol was added to samples in equal volumes and allowed to react for 30 min in the dark, the absorbance was recorded at 517 nm. The ABTS^+^ and DPPH scavenging capacity was calculated with the help of the following equation,

Free radical scavenging capacity (%) = (A_Control_ − A_Sample_)/A_Control_ × 100 (3)

where A_Control_ and A_Sample_ are the absorbances of the free radical solution without and with samples, respectively.

### 2.6. Functional Characteristics of Particles 

#### 2.6.1. Photochemical Stability of Quercetin

Photochemical stability of pristine and encapsulated quercetin was evaluated by the procedure presented by Sun et al. [26]. Quercetin dispersed in water and encapsulated in HZ and HZ-chi/pec particles at 5 µg/mL were irradiated up to 120 min with a 365 nm ultraviolet lamp (VWR International Inc., West Chester, PA, USA). Samples were collected at 0, 15, 30, 60, 90 and 120 min, and the content of quercetin was analyzed with the help of UV-Vis spectrophotometer mentioned above in Section 2.3.2.

#### 2.6.2. Storage Stability of Quercetin and Particles

Samples were stored at 25 and 45 °C for 28 days inside an LRH-250F incubator (Yiheng Scientific Instrument Co., Ltd. Shanghai, China). The stability of quercetin-loaded particles was analyzed in terms of particle size and ζ-potential during storage. The retention of quercetin was expressed as percent retention and calculated by using the following equation:

Quercetin retention (%) = Q_t_/Q_i_ × 100 (4)

where Q_i_ and Q_t_ are the content of quercetin at the beginning and specific time intervals during storage.

### 2.7. Statistical Analysis

All experiments were done in triplicates. The results were expressed in mean plus standard deviation and analyzed for a significant difference (*p* < 0.05) with IBM SPSS statistics 20.0 software package (IBM, Armonk, NY, USA).

## 3. Results and Discussion

### 3.1. Characterization of Hollow Zein Particles

#### 3.1.1. Effect of Na_2_CO_3_ and Chitosan on Hollow Zein Particles

**Figure 1:**
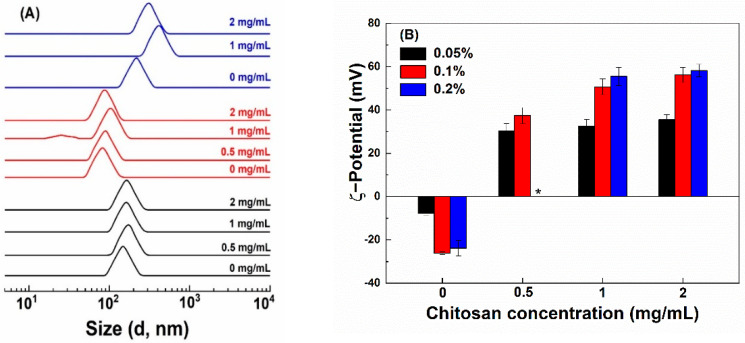
Particle size distribution (**A**) and ζ−potential (**B**) of hollow zein particles fabricated with 0.5% (black), 1% (red) and 2% (blue) Na2CO3 and coated with chitosan at various concentrations (Mark at the right). * indicates unstable particles.

The size of hollow zein particles is greatly influenced by the concentration of Na_2_CO_3_ used for the preparation of sacrificial templet [1,27]. The smallest particles of 76 nm were fabricated with 1% Na_2_CO_3_ (Figure 1A). At 0.5 and 2% Na_2_CO_3,_ the size of HZ particles was 145 and 210 nm, respectively. The reason for the bigger particles is due to the formation of a thicker zein shell around all the available sodium carbonate nuclei formed in ethanolic conditions at a Na_2_CO_3_ concentration of 0.5% but the formation of bigger Na_2_CO_3_ nanocrystal in size due to excessive aggregation and crystal growth at 2% [27]. The pH values of the HZ particle dispersions prepared with 0.5, 1 and 2% Na_2_CO_3_ were 9.04, 10.31 and 10.77, respectively, which is above the pI of zein [28]. The ζ-potential of HZ particles was −8, −26 and −24 mV when prepared with 0.5, 1 and 2% Na_2_CO_3_ (Figure 1B), receptively. There was a slight increase in the particle size of HZ particles upon the addition of chitosan (Figure 1A), except that precipitation was observed at 0.05% chitosan and 2% Na_2_CO_3_. The increase in the particle size was the most obvious when the chitosan concentration was 0.1% at 1% and 2% Na_2_CO_3_. In the presence of chitosan, the pH of particle dispersions shifted to 4.0–4.5. Chitosan interacts with proteins below their pI through hydrophobic, electrostatic, van der Waals and hydrogen bonding [12,29]. The ζ-potential of HZ-chi particles ranged between +30 and +58 mV (Figure 1B). The amine groups in chitosan contribute to the positive surface charge of composite zein particles [30,31]. These results suggest the formation of a chitosan shell.

#### 3.1.2. Fabrication of Pectin-Coated Composite Particles

HZ-chi particles prepared with 1% Na_2_CO_3_ were further coated with pectin to form a second layer. The addition of pectin did not influence particle PDI (Table 1). When the concentration of chitosan was 0.5 mg/mL, the size of HZ-chi particles increased from 87 to 170 and 180 nm upon adding 0.01 and 0.025 mg/mL of pectin, respectively. When the concentration of chitosan was 2 mg/mL, the size of HZ-chi particles increased from 84 to 194 and 192 nm upon adding 0.01 and 0.025 mg/mL of pectin, respectively. Meanwhile, a reduction in the ζ-potential positive values was observed, showing insufficient pectin to fully cover the surface of particles. With further increasing the pectin concentration to 0.05 and 0.1 mg/mL in the presence of 0.5 mg/mL chitosan and to at 0.05 mg/mL pectin in the presence of 2 mg/mL, the particle dispersion became unstable with the formation of precipitates, due to charge neutralization [7]. The neutralization was observed at 0.01 and 0.025 mg/mL pectin in the presence of 1 mg/mL chitosan (Table 1). However, in the presence of 1 mg/mL chitosan, an increment in size to 248 and 219 nm was observed at 0.05 and 0.1 mg/mL pectin, respectively, and ζ-potentials changed to negative values, suggesting the formation of pectin surface layer. An absolute value of ζ-potential above 20 mV is so high enough to ensure the physical stability of biopolymer-based particles [32]. Therefore, the hollow zein particles prepared with 1 mg/mL chitosan and 0.1 mg/mL pectin were used for further study on their physicochemical and functional attributes.

#### 3.1.3. Encapsulation of Quercetin 

Quercetin was used as a model bioactive flavonoid to be encapsulated in hollow zein particles. The loading efficiencies of quercetin were 72.71–79.86% in HZ particles, while the loading efficiencies increased to 90.58–93.86% at the flavonoid concentrations of 100–200 μg/mL and 85.84% at 250 μg/mL in HZ-chi particles (Table 2). The higher loading efficiency of quercetin in the presence of chitosan is attributed to its interaction with chitosan through electrostatic and hydrogen bonding, in addition to hydrophobic interaction with zein [33,34]. The loading capacity of quercetin in the absence and presence of chitosan remained similar and increased from about 2% to 6%. This is different from a decreasing trend that was commonly observed in composite protein particles with increasing the content of the wall materials [35,36].

**Figure 2:**
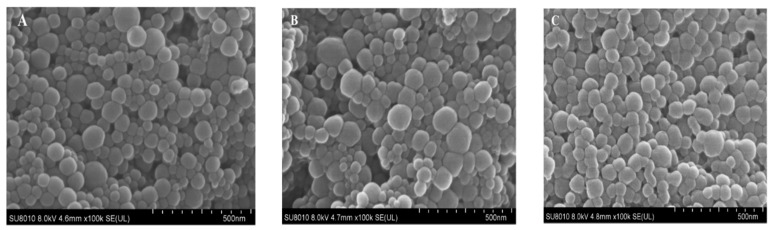
SEM images of quercetin-loaded hollow zein (qHZ, (**A**)), zein-chitosan (qHZ-chi, (**B**)) and zein-chitosan/pectin (qHZ-chi/pec, (**C**)) particles. The concentrations of chitosan and pectin were 1 and 0.1 mg/mL, respectively.

SEM images showed that quercetin-loaded HZ, HZ-chi and HZ-chi/pec were spherical with a smooth surface (Figure 2). The loading of quercetin increased the size of HZ particles without and with chitosan and/or pectin coating (Figure S1A). A similar trend was previously reported for the encapsulation of resveratrol and curcumin in composite hollow zein particles [11,37]. The loading of quercetin did not affect the ζ-Potential of HZ particles but increased ζ-potential absolute values of HZ-chi and HZ-chi/pec particles (Figure S1C), possibly due to rearrangement and exposure of more charged groups upon flavonoid inclusion [33]. 

### 3.2. Physical Characterization

#### 3.2.1. XRD

**Figure 3:**
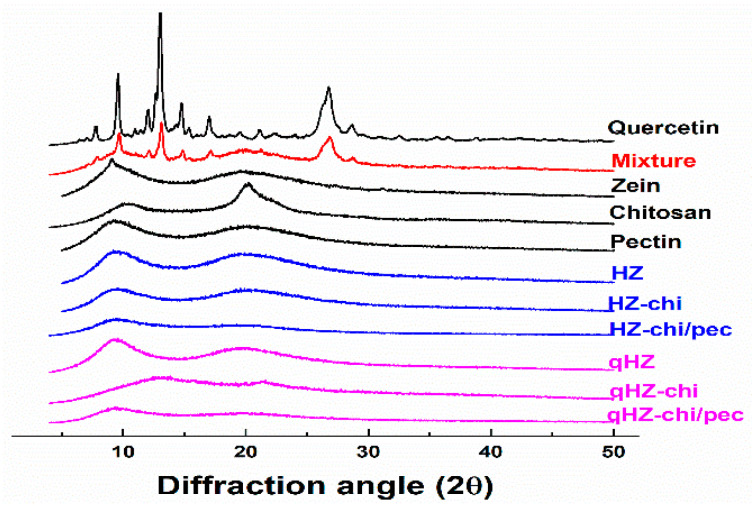
XRD diffraction patterns of quercetin, mixture (quercetin, zein, chitosan and pectin), zein, chitosan, pectin, HZ, HZ-chi, HZ-chi/pec, qHZ, qHZ-chi, qHZ-chi/pec.

Quercetin is a crystalline solid in its pristine form [38], as evident from sharp X-ray diffraction patterns at 8, 10, 12, 13.01, 15, 17 and 27 of diffraction angle (2θ) (Figure 3). The distinct peaks of quercetin with lower intensities were apparent in its mixture of zein, chitosan and pectin. In the diffractograms of qHZ, qHZ-chi and qHZ-chi/pec particles, the crystalline peaks of quercetin disappeared, advocating the transformation from crystalline to an amorous state upon encapsulation in particles [10,39]. Pure zein and chitosan showed mild crystallinity with moderate peaks at 9.56 and 20.24 2θ because of respective α-helixes and crystal lattice structure [40,41]. When zein was structured into HZ, HZ-chi, HZ-chi/pec, qHZ, qHZ-chi and qHZ-chi/pec particles, the diffraction pattern raised from α-helixes in zein and crystal lattice of chitosan became flattened due to interaction between the polymers during the formation of the composite particles [42,43].

#### 3.2.2. IR Spectroscopy

**Figure 4:**
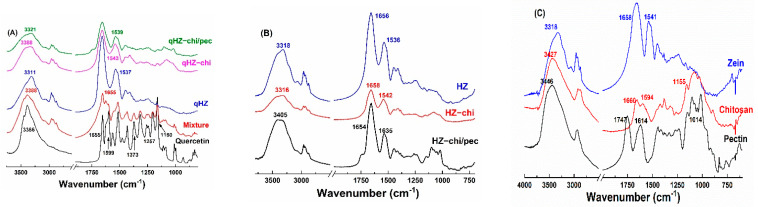
IR spectra of quercetin, mixture (quercetin, zein, chitosan and pectin), quercetin-loaded HZ−chi and HZ−chi/pec particles (**A**), HZ, HZ−chi and HZ−chi/pec blank particles (**B**), and raw materials zein, chitosan and pectin (**C**).

OH stretching of zein and HZ particles was at 3318 cm^−1^ but changed to 3316 and 3405 cm^−1^ in HZ-chi and HZ-chi/pec particles, indicating hydrogen bonding among zein, chitosan and pectin (Figure 4B,C). Likewise, amide I and II absorption band of zein changed from 1658 and 1541 cm^−1^ to 1656 and 1536 cm^−1^ in HZ particles, to 1658 and 1542 cm^−1^ in HZ-chi particles, and to 1654 and 1635 cm^−1^ in HZ-chi/pec particles. The variations of stretching and bending vibrations of C=O and N-H indicate that hydrophobic and electrostatic interactions occurred during the fabrication of HZ, chitosan and chitosan/pectin coated HZ particles [29]. The distinct absorption peaks of quercetin IR spectrum at 3386, 1655, 1599, 1373, 1257 and 1160 cm^−1^ (Figure 4A) signify O-H, C=O, C=C, C-OH, C-O-C and C-OH (B ring), respectively [34,44]. The absorption bands were clearly seen with lower intensities in the physical mixture of quercetin, zein, chitosan and pectin (Figure 4A). However, the characteristic absorption bands of quercetin were invisible in HZ, HZ-chi and HZ-chi/pec particles, indicating the entrapment of quercetin in the particles and limited starching and bending of various bonds [42]. The encapsulation of quercetin resulted in the variation of OH stretching of HZ, HZ-chi and HZ-chi/pec particles (Figure 4A,B), suggesting the flavonoid interaction with wall materials [45]. Upon encapsulation of quercetin in hollow zein particle, the amide I peak of zein was unchanged, whereas the amide II showed a redshift to 1537, 1543 and 1539 cm^−1^ in HZ, HZ-chi and HZ-chi/pec particles (Figure 4A). These shifts in the amide II result from hydrophobic, electrostatic and hydrogen bonding of quercetin with wall materials [46,47]. 

### 3.3. Stability of Hollow Particles

#### 3.3.1. Salt Endurance

**Figure 5:**
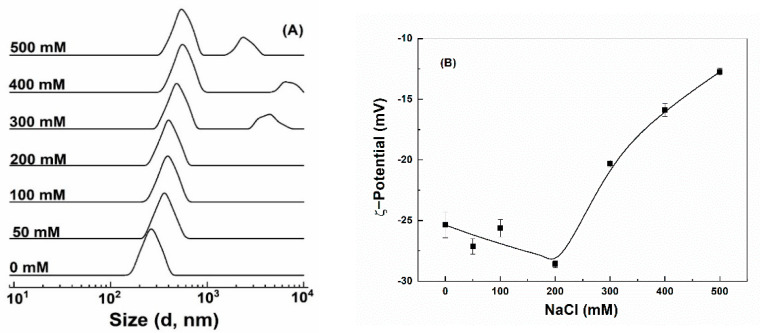
Size distribution (**A**) and ζ−potential (**B**) of quercetin−loaded hollow zein particles coated with chitosan and pectin as a function of NaCl concentration.

Colloidal stability against salt was assessed by exposing particles to various concentrations of NaCl. Quercetin-loaded hollow zein particles without and with chitosan were highly unstable and precipitated out even at 50 mM NaCl (Figure S2A,B), attributed to the screening effect and charge neutralization by counterions [7,43]. Quercetin-loaded HZ-chi/pec showed a remarkable endurance to precipitation up to 500 mM NaCl (Figure S2C). The size of quercetin-loaded HZ-chi/pec particles kept a single peak and increased gradually as the concentration of NaCl increased up to 200 mM (Figure 5A). Meanwhile, their ζ-potential changed to −29 mV (Figure 5B). It is possible that the NaCl ions screen the repulsion among the polysaccharide chain, leading to a greater adsorption of pectin on the particle surface [48,49]. These results are different from the sedimentation of pectin-coated zein particle at 70 mM NaCl reported by Huang et al. [16]. It can be thus speculated that the complex coating of chitosan and pectin provided better stability against salt than did by pectin alone. The size of quercetin-loaded HZ-chi/pec particles became bigger and had two peaks (Figure 5A), and their ζ-potential absolute values significantly decreased upon further increasing the concentration of NaCl. These results suggest the salt at high concentrations reduced the electrostatic repulsion between particles, resulting in their aggregation [46]. 

#### 3.3.2. pH Stability

**Figure 6:**
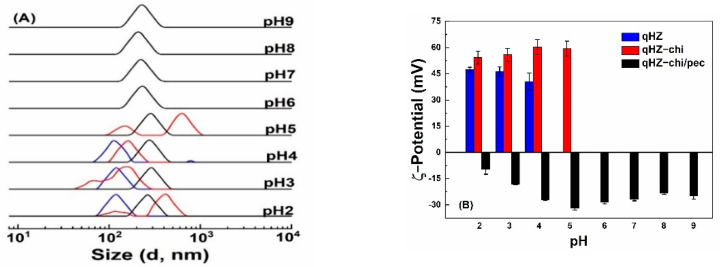
Size distribution (**A**) and ζ−potential (**B**) of quercetin−loaded hollow zein particles without (blue) and with chitosan (red) and chitosan/pectin (black) coating at various pH values.

Protein-based particles are liable to aggregation around pI [6], limiting their application. Quercetin-loaded hollow zein particles were sable with similar size distribution at pH 2, 3 and 4 (Figure 6A) but precipitated at pH 5 around pI, due to charge neutralization and lack of repulsive forces [50,51]. It is difficult to re-disperse the precipitated zein particles above the pI of zein. Quercetin-loaded hollow zein-chitosan particles showed two size distributions above and below pH 4 (Figure 6A). The change in the particle size below pH 4 is attributed to swelling and dissolution of the polymers with higher charge density [18], while the formation of bigger aggregates is at pH 5, close to the pI of zein [7]. The particles with chitosan were unstable at pH 6–9 due to the deprotonation of chitosan amine groups at pk_a_ and above [52,53]. It is noted that quercetin-loaded HZ-chi/pec particles showed good stability across the investigated pH range of 2–5 (Figure 6A). The ζ-potential of quercetin-loaded HZ-chi/pec was the highest at pH 5 and decreased as pH decreased (Figure 6B), due to deionization of the carboxylic groups of pectin below its pk_a_ (3.5) [16]. At pH 6–9, the size of quercetin-loaded HZ-chi/pec particles was around 220 nm and less than those at lower pH. Their ζ-potential also decreased slightly as the pH increased from pH 5. Karim et al. also reported a decline in the ζ-potential and size of pectin/chitosan-coated nanoliposome at pH 8 compared with that of pH 5 [18]. These changes might be due to the detachment of loosely adsorbed pectin molecules from the surface, since chitosan possesses less charged groups as the pH increases [54]. These findings suggest that the double coating with chitosan and pectin provides excellent protection against aggregation, especially around the pI of zein and pk_a_ of chitosan.

#### 3.3.3. Temperature Stability

**Figure 7:**
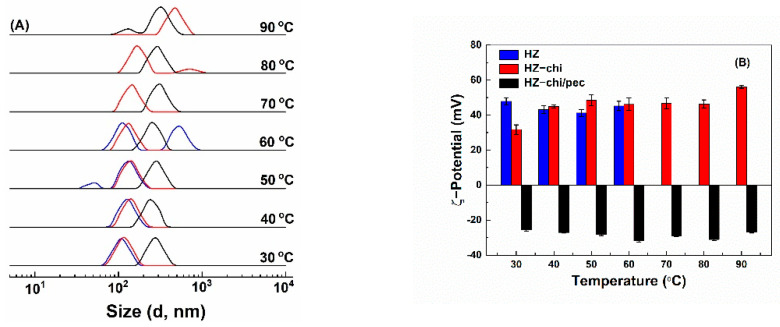
Effect of temperature on size distribution qHZ (blue), qHZ−chi (red) and qHZ−chi/pec (black) (**A**) and ζ−potential (**B**) of quercetin−loaded composite hollow zein particles.

Bioactive-component-loaded carrier systems for food application might be exposed to different temperature treatments during the production cycles. Figure 7 shows the effect of temperature on the size distribution and ζ-potential of quercetin-loaded HZ, HZ-chi and HZ-chi/pec particles. Quercetin-loaded HZ particles had ζ-potential values between +41–+48 mV (Figure 7B). Size of quercetin-loaded HZ particles increased as temperature increased and showed two peaks above 40 °C (Figure 7A), possibly attributed to rearrangement of zein molecules and exposure of nonpolar groups followed by a collapse of hollow structure [55]. This is different from solid zein particles, those were colloidally stable when heated at pH 4 and 80 °C for 120 min [43]. Likewise, Figure 7A shows that size of quercetin-loaded HZ-chi particles increased with increasing temperature, but two peaks were observed above 70 °C, suggesting that the chitosan coating improves the particle stability against heat treatment. Their ζ-potential was +32 mV at 30 °C and increased to a range of +45 and +48 mV at 40–80 °C and to +56 mV at 90 °C. The changes might be due to that the realignment of zein and chitosan at higher temperatures possibly leads to inter/intramolecular interactions [5,56]. The quercetin-loaded HZ-chi/pec particles were stable when the temperature was increased to 40 °C (Figure 7A). Then, their size gradually increased upon further increase in temperature and showed two peaks at 90 °C. Their ζ-potential was kept between −25 and −32 mV at all the investigated temperatures. These results indicate that the pectin coating further improves the colloidal stability of quercetin-loaded HZ-chi particles. It is presumed that steric stabilization of pectin coating inhibits the collision of zein particles with more exposed reactive functional groups at the higher temperature, thus preventing increment in size and particle aggregation [57].

#### 3.3.4. Storage Stability

**Figure 8:**
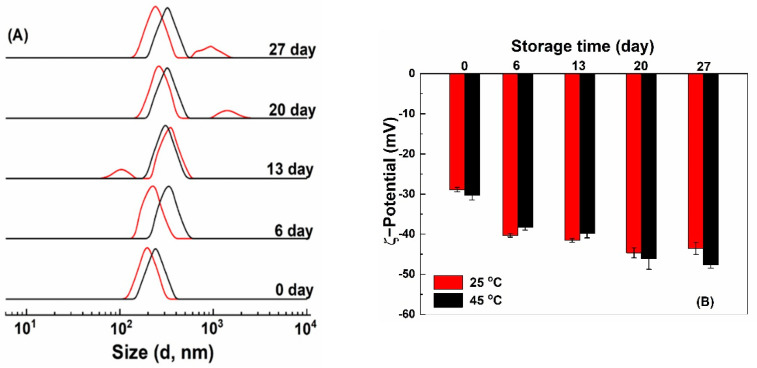
Size distribution (**A**) and ζ−potential (**B**) of quercetin−loaded hollow zein particles coated with chitosan and pectin at 25 °C (red) and 45 °C (black) during storage.

After storage at 25 and 45 °C for 7 days, quercetin-loaded HZ and HZ-chi particles precipitated, while no precipitation was observed for quercetin-loaded HZ-chi/pec particles (Figure S3). Figure 8 shows size distribution and ζ-potential of quercetin-loaded HZ-chi/pec particles during storage. Their size distribution remained a single peak during storage at 45 °C and increased to around 325 nm after 6 days. At 25 °C, the size distribution kept a single peak around 220 nm after 6 days and then showed two peaks. These results indicate quercetin-loaded HZ-chi/pec particles are more stable at 45 °C than 25 °C, possibly due to the high temperature facilitates adsorption of pectin to the particles more effectively and improves its complex formation capacity [58,59]. At 25 °C, a smaller size, around 90 nm was observed after storage for 13 days (Figure 8), probably due to the detachment, depolymerization and hydrolysis of galacturonic acid glycan chains of pectin at pH < 5 [60]. At 25 °C, a larger size around 900–1500 nm was observed after 20 days (Figure 8). The ζ-potential of HZ-chi/pec particles became more negative over time, both at 25 and 45 °C. These changes indicate the realignment of pectin during storage and exposure of more charged COO^-^ groups at the interface, leading to an upsurge in ζ-potential [18,54]. Pectin coating substantially enhances the colloidal stability of quercetin-loaded HZ-chi/pec compared with that of qHZ and qHZ-chi (Figure S3A,B) by preventing aggregation of particles through electrostatic repulsions and steric stabilization as well [61]. 

### 3.4. Antioxidant Activity

**Figure 9:**
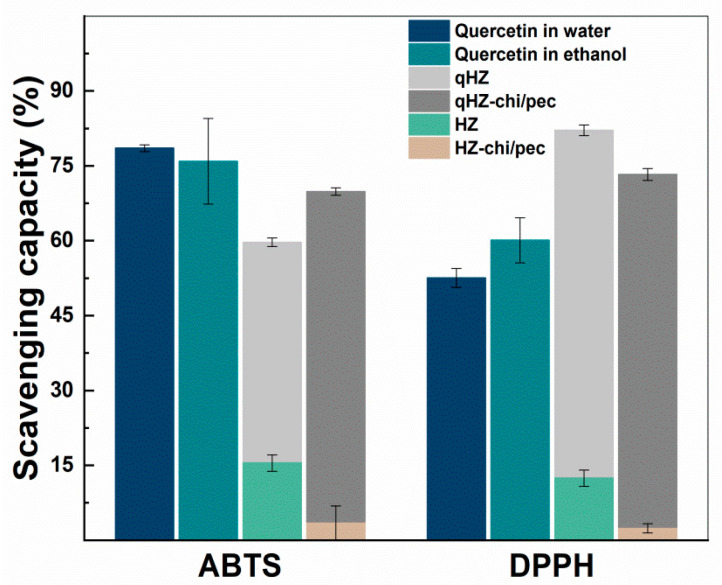
ABTS^+^ and DPPH scavenging capacity of quercetin, blank and quercetin-loaded HZ and HZ-chi/pec particles.

The antioxidant activity of encapsulated quercetin was investigated by ABTS and DPPH radical scavenging assays (Figure 9). The ABTS^+^ scavenging capacity of quercetin-loaded HZ was lower than the quercetin dispersed in water and ethanol. The reason for the lower scavenging capacity of encapsulated quercetin is being embedded in the hydrophobic pockets of protein and inaccessible to ABTS^+^ [30]. Furthermore, the hydroxyl groups in the B ring of quercetin are the main contributor of H^+^ and are involved in hydrogen bonding in the composite particles, thus unavailable to scavenge the free radicals. Earlier, quercetin encapsulated in SPI and solid zein particles showed a similar reduction in the ABTS^+^ savaging capacity. On the other hand, the interfacial chitosan-pectin coating in quercetin-loaded HZ-chi/pec particles may increase the accessibility of hydrophilic ABTS^+^ to quercetin, demonstrating higher scavenging of ABTS^+^ compared with that of qHZ (Figure 9). DPPH scavenging capacity of quercetin was improved upon encapsulation in HZ and HZ-chi/pec particles by compared with that of dispersed in ethanol and water (Figure 9). Earlier, it has been reported that both zein and DPPH being soluble in ethanol–water binary medium facilitate scavenging of DPPH by hydrophobic antioxidants encapsulated in the zein particles [62,63]. The ABTS and DPPH radical scavenging assay revealed that by encapsulating quercetin in composite hollow zein particles, it could be better protected from free reactive radicals in the surrounding medium along with an improved or sustained antioxidant activity.

### 3.5. Stability of Quercetin

#### 3.5.1. Photochemical Stability

**Figure 10:**
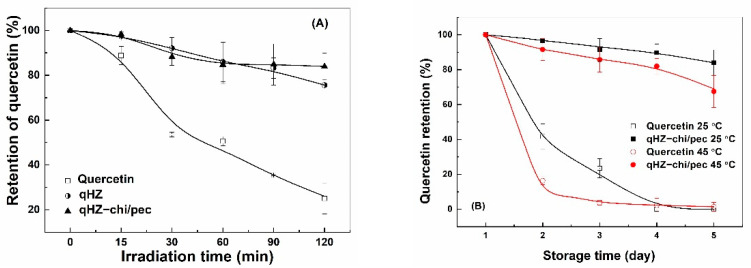
Retention of quercetin free and encapsulated in hollow zein particles coated without (qHZ) and with (qHZ-chi/pec) 1 mg/mL chitosan and 0.1 mg/mL pectin under irradiation (**A**) and during storage at 25 °C and 45 °C (**B**).

Quercetin is prone to degradation upon exposure to ultraviolet light due to oxidative decarboxylation of the C ring [22]. The retention of unencapsulated quercetin sharply decreased to 25% after 120 min of irradiation (Figure 10A). The quercetin loaded in HZ and HZ-chi/pec particles was more stable, with retention of about 80% after 120 min of irradiation. These results suggesting the hollow particle provide the excellent stability of quercetin against ultraviolet light. The protection results from the physical barrier and light-scattering effect of the particles [61]. The similar retention of quercetin in HZ and HZ-chi/pec particles (Figure 10A) indicates that the protection was fundamentally attributed to zein. A comparable protective effect has previously been reported by encapsulating quercetin in WPI/lotus root amylopectin, pea protein-isolated and zein/soluble soybean polysaccharide composite nanoparticles due to hydrogen and hydrophobic interaction between quercetin and proteins [39,43,64].

#### 3.5.2. Storage Stability 

Liu and coworkers reported a drastic decrease in the content of quercetin to around 38% in 3 days of storage at room temperature [39]. The degradation of quercetin was faster at 45 °C than 25 °C, with the retention of quercetin being 42% and 16% after 2 days (Figure 10B), respectively. Its complete loss was observed at 25 °C and 45 °C after 4 days. The degradation of quercetin is attributed to its auto-oxidation in the aqueous medium, which is much pronounced at elevated temperature [19,65]. The retention of quercetin was significantly greater in HZ-chi/pec particles during storage, being 84% and 67% at 25 and 45 °C after 27 days (Figure 10B), respectively. The ABTS^+^ scavenging capacity of quercetin-loaded HZ-chi/pec particles was lower than quercetin alone (Figure 9), indicating that the entrapment of quercetin reduces its accessibility to ABTS^+^ [30]. It is thus speculated that the entrapment in HZ-chi/pec particles (Table 2) provides a physical barrier between quercetin and environment-sensitive factors, resulting in the improved stability of quercetin (Figure 10B). Moreover, the hydrophobic interaction and hydrogen bond of quercetin with excipient biopolymer (Figure 4) may inhibit the flavonoid autoxidation and prolong its storage life.

## 4. Conclusions

The hollow zein particles coated with chitosan and pectin were prepared with 1% Na_2_CO_3_ as a sacrificial template. The hollow particles coated with 1 mg/mL chitosan and 0.1 mg/mL pectin had a size of 219 nm and ζ-potential of −28 mV. Chitosan coating improved the loading efficiency of quercetin in hollow zein particles. The coating of chitosan/pectin improved the stability of quercetin-loaded hollow zein particles against heat treatment, pH variation and salt. The entrapment in the hollow particles improved the photostability and storage stability of quercetin. The storage stability was better at 25 °C for entrapped quercetin but at 45 °C for hollow zein particles coated with chitosan and pectin. These findings will extend the application of composite hollow zein particles for the incorporation of bioactive components in functional products.

## Figures and Tables

**Table 1:** Effect of pectin on size, PDI and ζ-potential of hollow zein particles coated with chitosan.

Chitosan Concentration (mg/mL)	Parameter		Pectin Concentration (mg/mL)			
		0	0.01	0.025	0.05	0.1
0.5	Size (nm)	87 ± 2 ^Aa^	170 ± 1 ^Ab^	180 ± 2 ^Ac^	--	--
	PDI	0.21 ± 0.02 ^Aa^	0.20 ± 0.02 ^Aa^	0.21 ± 0.02 ^Aa^	--	--
	ζ-Potential (mV)	+37 ± 4 ^Aa^	+33 ± 1 ^Aa^	+26 ± 0 ^Ab^	--	--
1	Size (nm)	82 ± 2 ^Aa^	--	--	248 ± 4 ^Ac^	219 ± 1 ^Ab^
	PDI	0.12 ± 0.01 ^Ba^	--	--	0.18 ± 0.08 ^Aa^	0.12 ± 0.013 ^Aa^
	ζ-Potential (mV)	+51 ± 4 ^Ba^	--	--	−18 ± 0 ^Ac^	−28 ± 1 ^Ab^
2	Size (nm)	84 ± 2 ^Aa^	194 ± 5 ^Ab^	192 ± 4 ^Ab^	--	300 ± 6 ^Bc^
	PDI	0.18 ± 0.01 ^Aa^	0.14 ± 0.05 ^Aa^	0.11 ± 0.05 ^Ba^	--	0.13 ± 0.07 ^Aa^
	ζ-Potential (mV)	+56 ± 4 ^Ba^	+21 ± 1 ^Bb^	+22 ± 2 ^Bb^	--	−32 ± 2 ^Bc^

Values with different letters (upper case A and B for the concentration of chitosan; lower case a, b and c for the concentration of pectin) are significantly different in rows (*p* < 0.05); -- represents the aggregation followed by precipitation of nanoparticles.

**Table 2:** Loading efficiency and loading capacity of quercetin in hollow zein and zein-chitosan particles.

Quercetin Concentration (µg/mL)	Loading Efficiency (%)		Loading Capacity	
	Zein	Zein-Chitosan	Zein	Zein-Chitosan
100	79.07 ± 4.12 ^Aa^	93.86 ± 1.19 ^Ab^	2.59 ± 0.56 ^Ca^	2.22 ± 0.20 ^Ca^
150	75.72 ± 2.61 ^Aa^	91.36 ± 0.80 ^Ab^	3.92 ± 0.85 ^BCa^	3.76 ± 0.24 ^Ba^
200	79.86 ± 5.18 ^Aa^	90.58 ± 2.02 ^Ab^	5.69 ± 0.74 ^ABa^	5.53 ± 0.47 ^Aa^
250	72.71 ± 3.82 ^Aa^	85.84 ± 4.16 ^Bb^	6.29 ± 0.98 ^Aa^	5.89 ± 1.08 ^Aa^

Values with different letters (upper case A, B and C for the concentration of quercetin in column; lower case a and b in the row for the type of particles) are significantly different (*p* < 0.05).

## Data Availability

The data presented in this study are available in this manuscript.

## Supplementary Materials

The following are available online at https://www.mdpi.com/article/10.3390/antiox10091476/s1, Figure S1: Size distribution (A) and ζ-potential (B) of hollow zein (HZ) particles coated with chitosan (HZ-chi) and with chitosan and pectin (HZ-chi/pec) without and with quercetin. Figure S2: Visual appearance of quercetin-loaded hollow zein particles (A) coated with chitosan (B) and with chitosan and pectin (C) at 50–500 mM NaCl. Figure S3: Visual appearance of qHZ, qHZ-chi, qHZ-chi/pec at 25 °C (A), 45 °C (B). Figure S4: Calibration curve of quercetin.
