# Supplementary material for: Improving Physicochemical Stability of Quercetin-Loaded Hollow Zein Particles with Chitosan/Pectin Complex Coating
Source: Antioxidants (Basel). 2021 Sep 16;10(9):1476. doi: 10.3390/antiox10091476 (PMC8470427; doi:10.3390/antiox10091476)
Supplement: Supplementary file 1 [file antioxidants-10-01476-s001.zip › antioxidants-1375662-supplementary.pdf]

Supplementary Materials:

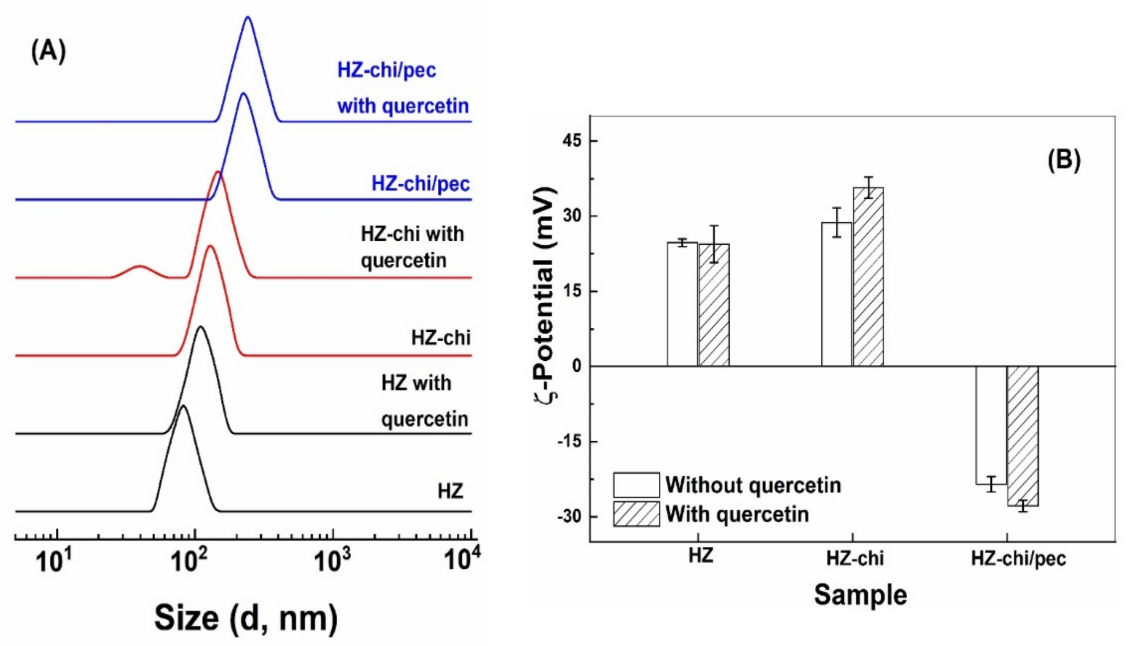

Figure S1. Size distribution (A) and ζ-potential (B) of hollow zein (HZ) particles coated with chitosan (HZ-chi) and with chitosan and pectin (HZ-chi/pec) without and with quercetin.

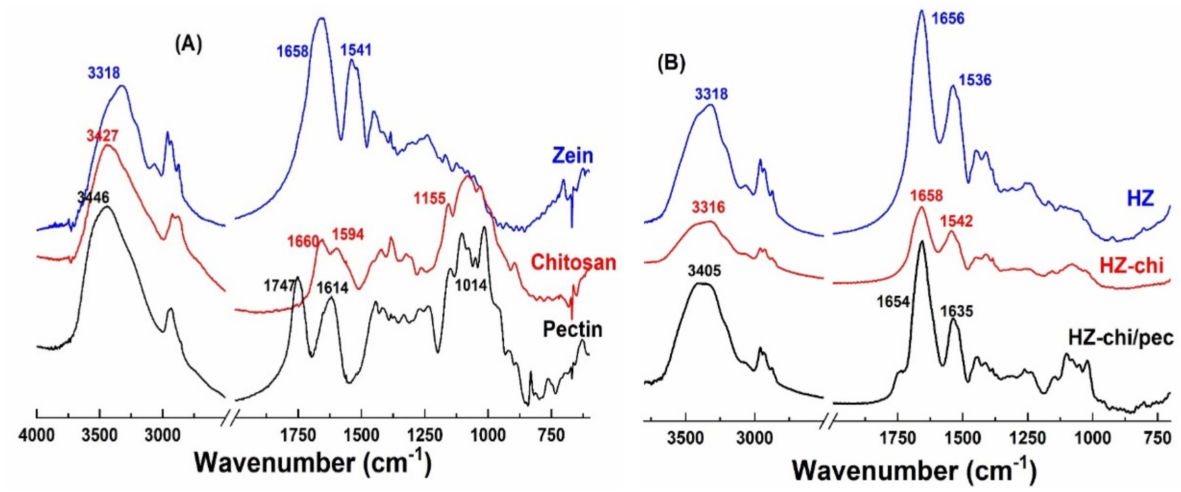

Figure S2. IR spectra of raw material (zein, chitosan and pectin) (A) and composite particles (HZ, HZ-chi and HZ-chi/pec) (B).

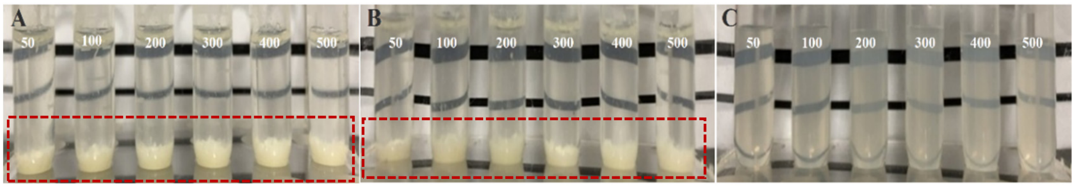

Figure S3. Visual appearance of quercetin-loaded hollow zein particles (A) coated with chitosan (B) and with chitosan and pectin (C) at 50 - 500 mM NaCl.

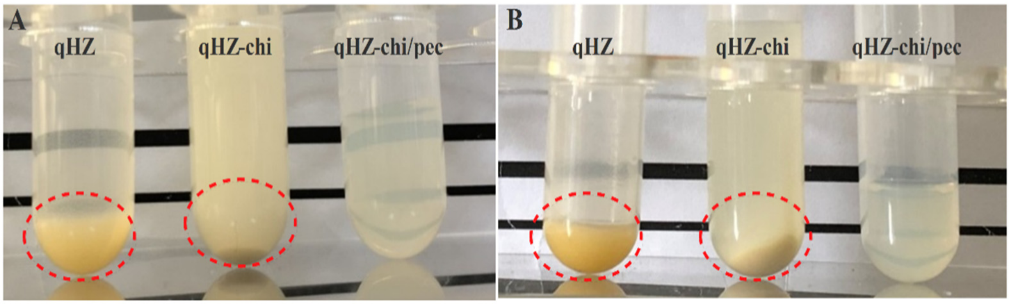

Figure S4. Visual appearance of qHZ, qHZ-chi, qHZ-chi/pec at 25°C (A), 45°C (B).
